# Diagnostic Value of Endotoxin Activity for Acute Postoperative Complications: A Study in Major Abdominal Surgery Patients

**DOI:** 10.3390/biomedicines12122701

**Published:** 2024-11-26

**Authors:** Hye Sung Kim, Gyeo Ra Lee, Eun Young Kim

**Affiliations:** Division of Trauma and Surgical Critical Care, Department of Surgery, Seoul St. Mary’s Hospital, College of Medicine, The Catholic University of Korea, Seoul 06591, Republic of Korea; dragon90@naver.com (H.S.K.);

**Keywords:** abdominal surgery, endotoxin activity, endotoxin activity assay, intensive care unit, liver function, predictive value, postoperative complications

## Abstract

**Background/Objectives**: Endotoxin, a component of lipopolysaccharide (LPS) from bacteria, disrupts the immune system, potentially leading to multiorgan failure. Unlike previous studies, we enrolled patients with mild clinical conditions after major abdominal surgery and assessed the predictive value of endotoxin activity (EA) levels for acute complications which occur within 7 days postoperatively. Also, the differential diagnostic value of EA was assessed in a subgroup of patients with abnormal liver function during the immediate postoperative period. **Methods**: Patients admitted to the surgical ICU of our institution following elective abdominal surgery were enrolled. Participants were classified into low/high postoperative EA groups based on EA cutoff values for predicting complications. Additionally, participants were categorized based on liver function assessed at ICU admission using total bilirubin (TB) levels. Abnormal liver function was defined as a TB level > 1.2 mg/dL. **Results**: 86 patients were analyzed. The EA cutoff for postoperative complications was 0.485, with 49 patients (57%) categorized in the low EA group (EA levels < 0.485) and 37 patients (43%) in the high EA group (EA levels ≥ 0.485). The high EA group experienced statistically worse outcomes, including longer ICU stays and higher mortality rates. Logistic regression analysis confirmed that EA levels and SOFA scores were significant predictors of postoperative complications. For patients with elevated TB, the EA cutoff value for postoperative complications was 0.515, which is higher than those obtained for the total patient cohort. **Conclusions**: EA level is a viable surveillance tool for detecting postoperative complications in the acute period among ICU patients undergoing major abdominal surgery, and must be interpreted carefully considering the patient’s liver function.

## 1. Introduction

Sepsis and septic shock continue to be the primary causes of mortality in intensive care units (ICUs), with sepsis accounting for over 10% and septic shock for more than 40% of in-hospital deaths despite advances in modern medicine [1,2]. Among the multifactorial etiologies of sepsis progression [3], a significant factor could be endotoxin. Endotoxin, a toxic component of lipopolysaccharide (LPS), which is predominantly found on the outer surface membrane of nearly all gram-negative bacteria, serves as a potent stimulant to the host’s immune system by binding to the immune cells or cell membranes of organs or tissues. Typically, endotoxin is released when bacteria die or their cell walls disintegrate, and excessive endotoxins prompt the patient’s immune system, leading to sepsis or septic shock [4,5].

Previous studies have attempted to accurately measure blood levels of endotoxin using the limulus amoebocyte lysate assay, the most widely employed method. Yet, this testing approach presents a substantial risk of contamination, and its reliability is compromised due to limulus reaction inhibitors [6,7,8]. An alternative, the endotoxin activity assay (EAA), assesses endotoxin levels by detecting chemiluminescence from neutrophil activity, providing highly reliable results within 30 min [6,7,9]. Previous research has demonstrated a correlation between increased endotoxin activity (EA) and more severe disease in patients with severe sepsis and septic shock [10], and our own study also indicated significantly higher in-hospital mortality in patients with septic shock after abdominal surgery when EA exceeded 0.54 [11]. However, most research involving EAA has been carried out in patients under highly clinically unstable conditions, such as shock or severe sepsis, with no studies on the prognostic value of EA for ICU patients in mild clinical conditions after major abdominal surgery. Major abdominal surgery is linked to a high risk of postoperative complications due to the extensive tissue damage and resultant inflammatory response. When infectious complications arise, they can swiftly lead to septic conditions and poor outcomes [12]. However, common symptoms such as fever, tachycardia, and inflammatory reactions during normal recovery can obscure the early detection of infectious complications. Thus, early identification and appropriate management of latent infections using EA may prevent progression to septic shock.

Herein, we assessed the predictive value of EA for infectious complications in the immediate postoperative period following major abdominal surgery. Given that endotoxin blood concentrations are likely abnormally high in patients with compromised liver function, owing to the hepatic reticuloendothelial system processing endotoxin in portal vein blood [9,13], we investigated the differential diagnostic value of EA in predicting complications among patient groups with abnormal liver function immediately post-operation.

## 2. Materials and Methods

### 2.1. Study Design and Participants

During November 2021 and September 2022, patients admitted to the surgical ICU of our institution following major abdominal surgery were eligible for study enrollment. The inclusion criteria were as follows: (1) Those who underwent major abdominal surgery under general anesthesia, irrespective of the type—laparoscopic, open, or robotic. ‘Major abdominal surgery’ is defined as an intra-peritoneal operation involving either luminal resection and/or solid organ resection associated with the gastrointestinal tract [14]. Both elective and emergency surgeries were included. (2) Those aged over 18 years. (3) Those capable of providing written consent. Exclusion criteria included: (1) patients with insufficient or missing test results for inflammatory markers, including EA, C-reactive protein (CRP), procalcitonin, and white blood cell (WBC) counts; (2) patients with a history of autoimmune disease or immunodeficiency; (3) patients prescribed immunosuppressive agents within the last 6 months; (4) patients with severe leukopenia (WBC count < 500 μL/L); (5) patients with uncontrolled active bleeding or unresolved sources of infection post-surgery; (6) patients with hematologic malignancy; or (7) pregnant patients. Patients were also excluded if they had agreed to a “do not resuscitate” order or refused any active resuscitation treatment. Subsequently, the enrolled participants were categorized into a low postoperative EA group (low EA group) and a high postoperative EA group (high EA group) based on EA cutoff values for postoperative complications. Additionally, participants were subgrouped based on their liver function assessed at ICU admission post-surgery. Regarding the pathophysiology of endotoxin, it enters the portal venous blood and is typically detoxified by Kuffer cells or excreted in bile in the liver [9,13,15]. In patients with impaired liver function, the detoxification process is disrupted, resulting in elevated endotoxin levels. Patients undergoing major abdominal surgery are susceptible to postoperative hepatic dysfunction primarily because of blood loss, tissue damage, or hypoperfusion during the surgical procedure [16,17]. Therefore, a detailed analysis of endotoxin trends and distinct predictive values of endotoxin levels in subgroups based on liver function would be clinically valuable. Liver function was assessed using total bilirubin (TB) levels measured from participants’ blood samples upon admission to the ICU postoperatively, and we subgrouped those with abnormal liver function defined as TB > 1.2 mg/dL. This study received approval and was monitored by our Institutional Review Board (IRB No. KC21TISI0611), and it was conducted in accordance with the Declaration of Helsinki and its subsequent amendments.

### 2.2. EA Measurement

The EAA was conducted using a murine immunoglobulin M monoclonal antibody against the lipid A of *Escherichia coli* J5, following the method described by Romaschin et al. [7]. The EA levels of all participants were measured at least once for each patient, immediately after surgery upon ICU admission, with a subset rechecked after 24 and 48 h. A separate 2 mL sample of whole blood was collected in EDTA tubes from study participants at the time of laboratory exam, during postoperative admission to the ICU. These blood samples were stored at 18–25 °C and analyzed within 1 h of collection. The detection of LPS involves the differential activation of polymorphonuclear leukocytes in antibody-containing tubes relative to control tubes. The formation of an LPS/anti-LPS complex primes the patient’s neutrophils, enhancing their response to zymosan stimulation. These neutrophils release oxidants, reacting with luminol to emit chemiluminescence, quantified as a ratio of sample to positive control, and measured on a scale from 0 to 1 (Autolumat LB 953; E. G. & G. Berthold, Wildbad, Germany). Samples were deemed appropriate if the coefficient of variation was below 15% for an EA level > 0.20 and below 30% for an EA level ≤ 0.20.

EAA levels are on a scale of 0 to 1, with 0–0.4 indicating low endotoxin activity, 0.4–0.6 indicating intermediate levels, and EAA > 0.6 indicating high levels.

### 2.3. Data Collection and Study Outcomes

For all participants, data were collected and reviewed retrospectively from operative records or medical charts from the time of study enrollment. The degree of organ dysfunction at ICU admission was assessed using the Sequential Organ Failure Assessment (SOFA) score, and the Acute Physiology and Chronic Health Evaluation-II (APACHE-II) score evaluated the initial severity of the patients’ illness. Data including the type and site of operation, operation duration, and estimated blood loss were obtained from the operative record. Operations were categorized and analyzed according to the site of target organ as upper gastrointestine (GI), lower GI, and hepatobiliary. Blood samples were collected from the participants immediately post-surgery upon ICU admission and underwent various laboratory tests and cultures. Laboratory exams covered diverse inflammatory markers such as CRP, procalcitonin, and WBC, and culture tests were conducted on samples including blood, sputum, surgical drainage fluid, and urine, every three days while in the ICU.

Sepsis or septic shock was diagnosed according to the Sepsis-3 guidelines, which define sepsis as life-threatening organ dysfunction caused by dysregulation of the host’s response to infection. Septic shock is a subset of sepsis that necessitates the use of vasopressors to maintain a mean arterial pressure (MAP) of 65 mmHg or higher, along with serum lactate levels exceeding 2 mmol/L after adequate resuscitation [18]. All patients diagnosed with sepsis were managed in accordance with the Sepsis Survival Campaign guidelines [19]. For persistent hypotension due to septic shock, 30 mL/kg of crystalloid solution was administered during fluid resuscitation, and vasopressors were employed if the MAP subsequently fell below 65 mmHg. Culture tests were conducted in the ICU upon admission, and antibiotics were administered empirically until test results were available. Once results were confirmed, the antibiotic strategy was adjusted in consultation with an infectious disease specialist, based on the bacterial type and its antibiotic susceptibility.

Postoperative complications are delineated as those occurring within 7 days of surgery and classified as grade 3 or higher based on the Clavien–Dindo classification. This category encompasses complications that necessitate endoscopic, radiologic, or surgical intervention, or those resulting in life-threatening conditions or death [20]. Mortality was meticulously reviewed and categorized into ICU mortality, 7-day mortality, 28-day mortality, in-hospital mortality, and overall mortality. ICU mortality is defined as deaths that occur during an ICU stay, while in-hospital mortality encompasses all deaths that occur within the same hospital stay. Overall mortality refers to deaths happening within 3 months of surgery.

### 2.4. Statistical Analysis

The primary outcome of the current study was the incidence of postoperative complications graded 3 or higher according to the Clavien–Dindo classification that occurred within 7 days following surgery. The secondary outcome was the 28-day mortality rate post-surgery. To establish the cutoff value of EA levels associated with postoperative complications, receiver operating characteristic (ROC) curve analysis was conducted, and patients were categorized into low and high EA groups based on postoperative EA levels. We compared demographics and clinical outcomes between these groups, which were delineated by EA cutoff values. Categorical variables were analyzed using either Fisher’s exact test or the Chi-squared (χ^2^) test. Continuous data were presented as mean ± standard deviation, with overall differences evaluated by either the Student’s *t*-test or analysis of variance. The normal distribution of variables was assessed using the Kolmogorov–Smirnov test, and variables not normally distributed were analyzed via the nonparametric Mann–Whitney test. Univariate logistic regression analysis explored the predisposing factors for postoperative complications or 28-day mortality, and only significant variables in univariate analysis were included in multiple regression analysis using the Cox proportional hazard model, accompanied by relative risk and a corresponding 95% confidence interval (CI). Differences were considered statistically significant with *p*-values < 0.05. For subgroup analysis, patients exhibiting abnormal liver function with a TB level increase of more than 1.2 mg/dL upon admission to the ICU post-surgery were selectively chosen, and the cutoff for postoperative complications occurrence was determined using ROC curve analysis. Subsequently, we segregated these subgroup patients into high and low EA groups, compared clinical outcomes between the two groups, and conducted multiple regression analysis to identify risk factors for postoperative complications or 28-day mortality post-surgery. All statistical analyses were performed using the SPSS statistical package software for Windows (version 24.0, SPSS Inc., Chicago, IL, USA).

## 3. Results

### 3.1. Study Population Selection and Group Classification

**Figure 1:**
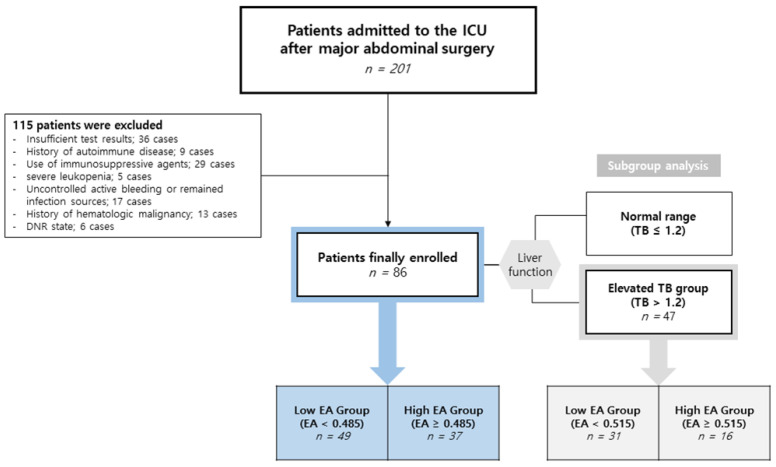
Schematic diagram of patient enrollment.

During the study period, 201 patients underwent major abdominal surgery under general anesthesia and were subsequently admitted to the surgical ICU. Of these, 115 patients were excluded based on our criteria: 36 due to insufficient test results, 9 with autoimmune disease, 29 using immunosuppressive agents, 5 with severe leukopenia, 17 with uncontrolled active bleeding or persistent infection sources postoperatively, 13 with hematologic malignancy, and 6 who signed a “do not resuscitate” order. Finally, 86 patients qualified for study analysis (Figure 1).

**Figure 2:**
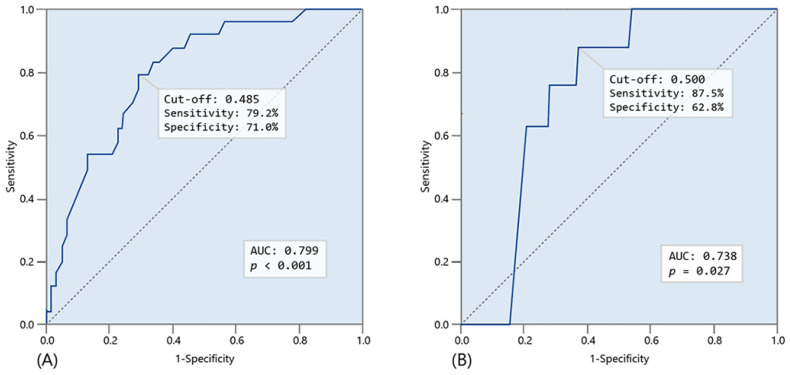
ROC curve analysis to determine cutoff value of endotoxin activity (EA) for clinical outcomes in total participants (**A**) for occurrence of postoperative complication and (**B**) for 28-day mortality after surgery.

As shown in Figure 2A, the cutoff value for EA level associated with postoperative complications was 0.485, with an area under the curve (AUC) of 0.799 {Sensitivity (Se) = 79.2%, Specificity (Sp) = 71.0%, *p* < 0.001}. Based on this EA cutoff, participants were divided into two groups: 49 in the low EA group (57%, EA levels < 0.485) and 37 in the high EA group (43%, EA levels ≥ 0.485).

### 3.2. Demographic and Clinical Data

Table 1 presents the demographic and disease profile comparisons between the groups. The average age was 62.4 years, with 52 (60.5%) being male, showing no significant difference between the groups. The mean SOFA score at ICU admission was 4.7, and the mean APACHE II score was 15.8; the high EA group had significantly higher scores in both measures. The most common surgical site was hepatobiliary (52 cases, 60.5%), followed by lower GI (31 cases, 36.0%). A total of 33 patients (38.4%) underwent emergency operations, with the high EA group experiencing more frequent emergencies. In baseline laboratory tests, the high EA group had significantly higher CRP levels (9.04 mg/dL vs. 1.6 mg/dL, *p* < 0.001), procalcitonin levels (35.27 ng/mL vs. 12.07 ng/mL, *p* = 0.024), and lactate levels (4.77 mmol/L vs. 2.39 mmol/L, *p* = 0.001).

### 3.3. Clinical Outcome and Risk Factor Analysis

Table 2 details the clinical outcome comparisons between the two groups based on postoperative EA levels. The high EA group exhibited significantly higher incidences of sepsis, septic shock, and postoperative complications compared to the low EA group. The high EA group also demonstrated worse outcomes in all mortality metrics, including ICU mortality (18.9% vs. 4.1%, *p* = 0.035), 28-day mortality (18.9% vs. 2%, *p* = 0.019), and overall mortality (18.9% vs. 4.1%, *p* = 0.035).

Table 3A summarizes the logistic regression analysis results for postoperative complications in patients who underwent major abdominal surgery. The univariate analysis revealed significant associations between EA levels, SOFA score, CRP, lactate, and postoperative complications. In the multivariate analysis, EA levels (OR: 300.516, 95% CI 16.149–5592.344, *p* < 0.001) and SOFA score (OR: 1.522, 95% CI 1.287–1.800, *p* < 0.001) emerged as significant predisposing factors for postoperative complications. We also generated a ROC curve for the EA cutoff level related to 28-day mortality, as shown in Figure 2B. The cutoff value was 0.5, higher than the 0.485 for postoperative complications, with an AUC of 0.738 (Se = 87.5%, Sp = 62.8%, *p* = 0.027). Table 3B reveals that EA levels, SOFA score, and lactate were significant factors in the univariate analysis of 28-day mortality, while multivariate analysis indicated that only the SOFA score (OR: 1.360, 95% CI 1.135–1.630, *p* = 0.001) was a significant risk factor for 28-day mortality post-surgery.

### 3.4. Subgroup Analysis of Elevated TB Group

**Figure 3:**
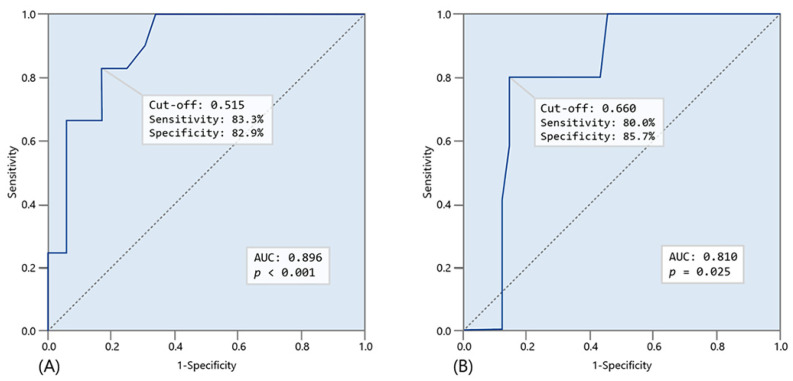
ROC curve analysis to determine cutoff value of endotoxin activity (EA) for clinical outcomes in elevated total bilirubin (TB) group participants (**A**) for occurrence of postoperative complication and (**B**) for 28-day mortality after surgery.

For subgroup analysis targeting patients with elevated TB values, we employed ROC curves to determine different EA cutoff values for clinical outcomes (Figure 3). The EA cutoff values were 0.515 for postoperative complications (AUC = 0.896, Se = 83.3%, Sp = 82.9%, *p* < 0.001) and 0.66 for 28-day mortality (AUC = 0.81, Se = 80%, Sp = 85.7%, *p* = 0.025), both exceeding those determined for the total patient cohort (Figure 3). We subdivided the patients into low and high postoperative EA groups and compared clinical outcomes between the two (Table 4). The elevated TB group consisted of 47 patients, segmented into a low EA group (n = 31, 66%, EA levels < 0.515) and a high EA group (n = 16, 34%, EA levels ≥ 0.515). Patients in the high EA group exhibited significantly higher rates of septic shock (56.3% vs. 6.5%, *p* < 0.001) and a prolonged ICU stay duration (9.8 days vs. 3.6 days, *p* = 0.038). Both the postoperative complication rates (62.5% vs. 6.5%, *p* < 0.001) and the 28-day mortality rates (25% vs. 3.2%, *p* = 0.040) were significantly higher compared to the low EA group.

Table 5 presents the logistic regression analyses for postoperative complications and 28-day mortality in the elevated TB group. In univariate analysis, EA levels, SOFA score, CRP, and lactate were significant factors for postoperative complications. However, multivariate analysis identified only EA levels (OR: 10,286.214, 95% CI 43.683–2,422,130.566, *p* = 0.001) as a significant risk factor. As for the 28-day mortality, the SOFA score and lactate were significant in univariate analysis, but no factors emerged as significant risks in multivariate analysis.

## 4. Discussion

In this study, patients admitted to the ICU with higher EA levels exceeding 0.485 following major abdominal surgery experienced significantly worse outcomes, including postoperative complications and mortality. Logistic regression analysis identified the EA level as a significant risk factor for postoperative complications along with the SOFA score, but not for mortality. Furthermore, in subgroup analysis of patients with hepatic dysfunction marked by elevated TB levels, the EA cutoff for postoperative complications was higher than that for the entire patient population. Additionally, EA was identified as the sole significant risk factor for postoperative complications in the elevated TB group.

Endotoxin plays a pivotal role in the pathophysiology of septic shock, serving as the main component of LPS, the most potent immune stimulant known to date. LPS activates Toll-like receptor-4, inducing inflammatory cytokine production and systemic inflammation [3]. When there is excessive activation of the host immune system, a cytokine storm associated with severe systemic inflammation response may occur, ultimately leading to profound immune disturbance and anergy. This results in a refractory shock state and multiorgan failure, which contribute to the high mortality rates observed in sepsis or septic shock patients [21]. Consequently, numerous studies have attempted to correlate EA level with disease severity and prognosis. Marshall et al. reported that a rapid increase in the EA level in patients suffering from severe sepsis or septic shock was significantly associated with increased disease severity [10]. Similarly, another study involving patients with systemic inflammatory response syndrome found that EA levels significantly increased in those suspected of sepsis with positive blood cultures, and these levels were strongly correlated with disease severity [22]. Our results also indicate that patients in the high EA group experienced more severe disease, characterized by worse clinical outcomes such as longer hospital stays, a higher incidence of complications, and increased mortality rates. Furthermore, elevated EA levels serve as predictors of postoperative complications, as demonstrated in both univariate and multivariate analyses. Distinguishing between clinical manifestations of the postoperative recovery process and symptoms associated with postoperative complications proves inherently challenging. During the postoperative period, patients might present non-specific symptoms such as increased heart rate or body temperature due to the inflammatory response provoked by the surgery. Normal postoperative inflammation can be mistaken for complications like peritonitis or sepsis caused by surgical site infections or intraabdominal leakage. This confusion complicates early diagnosis and delays appropriate management, thereby exacerbating the patient’s condition and leading to poorer outcomes. In particular, major abdominal surgery may induce complications such as anastomosis leakage in relatively fragile tissue, bleeding, or intraabdominal abscess, all of which are likely postoperative outcomes due to complex anastomosis formation in the gastrointestinal tract. Such complications can lead to panperitonitis or shock, resulting in critical outcomes. Although biomarkers like procalcitonin, CRP, and lactate have been suggested as potential diagnostic tools for distinguishing these complications, they have not yet demonstrated sufficient specificity. Specifically, procalcitonin is significantly elevated in bacterial infections and can be used as a marker, but its elevation in conditions such as rhabdomyolysis, severe pancreatitis, and some autoimmune diseases reduces its diagnostic specificity [23,24,25]. Likewise, while CRP is an acute-phase reaction material that increases with infection or inflammation, it can also rise due to other causes such as trauma, making it an unreliable sepsis severity indicator [23,25,26]. Our analysis revealed that conventional markers like CRP and lactate did not show statistical significance in a multivariate regression analysis of postoperative complications. However, a high EA level was identified as a significant factor, suggesting that EAA, which utilizes endotoxins closely related to the pathophysiology of inflammatory complications and sepsis, could help overcome the limitations of non-specificity in conventional markers. In this study, high EA levels were significant predictors of postoperative complications, and a higher incidence of infectious complications was observed in the high EA group. However, these findings did not achieve statistical significance, and when considered alongside the lack of significance in the multivariate analysis of 28-day mortality, this may be attributable to the overall low disease severity in the study population. Generally, patients were in good health before surgery, with more than half undergoing elective procedures and a low incidence of comorbidities such as heart failure, end-stage renal disease, or cardiovascular events. At ICU admission, the average SOFA and APACHE II scores were 4.7 and 15.8, respectively, reflecting relatively low disease severity. Subsequently, the 28-day mortality rate was low at 9.5% across the entire patient cohort, suggesting insufficient data to analyze risk factors associated with 28-day mortality. Therefore, further studies should be conducted to explore the relationship between EA levels and 28-day mortality in larger cohorts of patients with higher mortality rates. Nonetheless, as demonstrated in this study, EAA could be especially beneficial for patients undergoing major abdominal surgery, particularly those at high risk for postoperative complications.

Additionally, we conducted a sub-analysis to assess the correlation between EA level and prognosis in patients with diminished liver function. This is based on the observation that recent advances in surgical techniques have led to an increased number of surgeries in high-risk patients of advanced age or with multiple comorbidities, who often exhibit preoperative liver dysfunction. Also, intraoperative bleeding and the drugs used for anesthesia may contribute to a decrease in liver function, and patients with initial hepatic dysfunction are generally at a higher risk of postoperative hepatic complications or severe deterioration compared to those with normal liver function [27]. Particularly, major abdominal surgery is associated with a higher risk of postoperative hepatic impairment compared to other surgeries, due to the likelihood of significant bleeding or extended operation times. However, under conditions of impaired liver function, traditional biomarkers fail to accurately reflect the systemic condition of patients. In procalcitonin’s case, the release of damage-associated molecular patterns from hepatocytes following liver injury induces procalcitonin production, and elevated procalcitonin levels exacerbate cellular damage, perpetuating a harmful cycle [28]. In the case of lactate, clearance and normalization are usually compromised due to reduced liver function [29,30], complicating accurate assessment and hindering early detection of postoperative complications and appropriate monitoring. Conversely, the findings of this study indicate that elevated EA levels in patients with hepatic dysfunction could serve as an early indicator of postoperative complications. This suggests that EA levels could compensate for the limitations of traditional biomarkers in patients with compromised liver function after major abdominal surgery. Nevertheless, considering the metabolic processes of endotoxin in the body, patients with impaired liver function may need a distinct cutoff value compared to those with normal liver function. Endotoxins are primarily eliminated by the liver. During this process, a certain proportion of endotoxins in portal vein blood is absorbed by Kupffer cells, while the majority is transformed into bile by hepatocytes and then excreted [9,15]. Therefore, if this endotoxin excretion process is impeded by impaired liver function, EA levels may appear higher than they actually are. In a study by Chan et al., it was demonstrated that EA levels significantly increase as liver function worsens in patients with liver cirrhosis [13]. In the current results, the cutoff value for EA levels associated with postoperative complications was found to be higher in the elevated TB group (0.515) compared to all participants (0.485). Thus, EA levels should be interpreted in the context of liver function, with higher cutoff values recommended for patients with impaired liver function. If further research involving a larger population can accurately determine these cutoff values for EA levels, this insight could prove beneficial in predicting both the prognosis and the occurrence of postoperative complications in patients undergoing major abdominal surgery, particularly those with hepatic dysfunction.

Despite these interesting findings, the study has various limitations. First, the retrospective design limited the dataset, resulting in overlooked cases. In one example, alanine transaminase could have been used to assess liver function in addition to total bilirubin, but the current data do not include enough cases with data of alanine transaminase due to the limitation of being a retrospective study, so it could not be analyzed. Additionally, not all biomarkers were tested in certain instances, necessitating the exclusion of those cases when comparing EA levels with conventional markers such as presepsin and procalcitonin. This led to a reduced case number for analysis, potentially diminishing the statistical power of the primary results. Second, participants undergoing elective surgeries generally had less severe conditions and were typically in better preoperative states, consequently leading to lower rates of mortality and postoperative complications. This factor made achieving statistically significant differences challenging, particularly in the analyses of mortality and severe postoperative complications. A larger, more diverse prospective follow-up study is necessary to validate these findings. Third, the selection of EA cutoff values may vary in different clinical settings, particularly in more diverse patient populations or settings with higher rates of underlying comorbidities. These factors may influence the applicability of the cutoff values identified in this study to other patient groups, highlighting the need for further validation. Nevertheless, this research uniquely examines postoperative patients in general, including those from elective operations, whereas previous studies on EAA predominantly covered severe cases such as septic shock or sepsis. Moreover, EAA could serve as an effective monitoring tool for patients undergoing major abdominal surgeries prone to complications. In addition, the EA cut-off values for postoperative complications proposed in this study are within the intermediate range of EA. Identifying patients in the intermediate range provides an important opportunity to apply therapies that may improve prognosis, as there is limited benefit from treatment at very high EA levels and less need for treatment at low EA levels. In this respect, the cutoff values proposed in this study are reasonable from a clinical perspective and highlight the clinical importance of the intermediate range. Notably, our findings indicate that EAA could more precisely evaluate patients with impaired liver function than traditional biomarkers.

## 5. Conclusions

In conclusion, EA levels could be an effective monitoring tool for postoperative complications in surgical ICU patients after major abdominal surgeries. Precise interpretation of EA levels with consideration of the patient’s liver function is crucial.

## Figures and Tables

**Table 1:** Comparison of demographics and disease profiles between two groups according to postoperative endotoxin activity (EA) levels.

	All Patients	Low EA (EA < 0.485)	High EA (EA ≥ 0.485)	*p*-Value
	n = 86	n = 49 (57%)	n = 37 (43%)	
Demographic characteristics				
Age, years (mean ± SD)	62.4 ± 15.4	59.7 ± 16.1	66 ± 13.9	0.062
Sex, Male, n (%)	52 (60.5)	30 (61.2)	22 (59.5)	1
Body mass index, kg/m^2^ (mean ± SD)	23.46 ± 3.86	23.88 ± 3.91	22.91 ± 3.77	0.25
SOFA score at ICU admission (mean ± SD)	4.7 ± 4.7	2.7 ± 2.9	7.4 ± 5.3	<0.001
Respiratory	1.2 ± 1.4	0.7 ± 1.1	1.9 ± 1.4	<0.001
Cardiovascular	1.3 ± 1.9	0.4 ± 1.2	2.4 ± 2	<0.001
CNS	1.4 ± 1.9	0.2 ± 0.7	0.7 ± 1.1	0.015
Liver	0.9 ± 1	1 ± 1	0.7 ± 0.9	0.17
Renal	0.5 ± 1	0.2 ± 0.5	0.9 ± 1.2	0.002
Coagulative	0.4 ± 0.9	0.2 ± 0.6	0.8 ± 1.2	0.005
APACHE II score at ICU admission (mean ± SD)	15.8 ± 8.6	11.8 ± 5.7	19 ± 9.3	0.001
Underlying disease, n (%)				
Hypertension	41 (47.7)	21 (42.9)	20 (54.1)	0.384
Diabetes	21 (24.4)	14 (28.6)	7 (18.9)	0.325
Cerebrovascular accident	11 (12.8)	4 (8.2)	7 (18.9)	0.195
Heart failure	1 (1.2)	0	1 (2.7)	0.43
Chronic kidney disease	10 (11.6)	5 (10.2)	5 (13.5)	0.739
End stage renal disease	5 (5.8)	2 (4.1)	3 (8.1)	0.648
Operation record				
Operation time, min (mean ± SD)	241 ± 108.2	272.7 ± 105.4	199.7 ± 98.4	0.002
Estimated blood loss, mL (mean ± SD)	372.8 ± 343.3	393.6 ± 293.8	345.1 ± 402.3	0.52
Emergent operation	33 (38.4)	9 (18.4)	24 (64.9)	<0.001
Baseline laboratory findings				
WBC, ×10^9^ counts/L (mean ± SD)	11.91 ± 6.94	13.3 ± 6.49	10.07 ± 7.18	0.032
Hemoglobin, g/dL (mean ± SD)	11.63 ± 3.34	12.39 ± 3.88	10.63 ± 2.12	0.014
Platelet, ×10^9^ counts/L (mean ± SD)	166.7 ± 103.2	206.3 ± 85.6	114.3 ± 102.1	<0.001
Prothrombin time, % (mean ± SD)	72 ± 26.28	80.85 ± 21.71	60.27 ± 27.46	<0.001
BUN, mg/dL (mean ± SD)	20.06 ± 16.52	13.87 ± 6.3	28.25 ± 21.69	<0.001
Creatinine, mg/dL (mean ± SD)	1.35 ± 1.4	0.93 ± 0.5	1.9 ± 1.94	0.005
C-reactive protein, mg/dL (mean ± SD)	4.8 ± 7.95	1.6 ± 4.52	9.04 ± 9.47	<0.001
Procalcitonin, ng/mL (mean ± SD)	22.29 ± 38.94	12.07 ± 35.87	35.27 ± 39.46	0.024
Lactate, mmol/L (mean ± SD)	3.5 ± 3	2.39 ± 1.98	4.77 ± 3.46	0.001

EA, endotoxin activity; SOFA, sequential organ failure assessment; APACHE II, Acute Physiology and Chronic Health Evaluation-II; WBC, white blood cells; BUN, blood urea nitrogen.

**Table 2:** Comparison of clinical outcomes between two groups according to postoperative endotoxin activity (EA) levels.

	All Patients	Low EA (EA < 0.485)	High EA (EA ≥ 0.485)	*p*-Value
	n = 86	n = 49 (57%)	n = 37 (43%)	
Postoperative complications				
Anastomosis leakage	3 (3.5)	1 (2)	2 (5.4)	0.575
Bile leakage	2 (2.3)	0	2 (5.4)	0.182
Intraabdominal fluid collection	22 (25.6)	13 (26.5)	9 (24.3)	1
Pneumonia	10 (11.6)	3 (6.1)	7 (18.9)	0.092
Postop bleeding	3 (3.5)	1 (2)	2 (5.4)	0.575
Wound infection	0			
Arrhythmia	12 (18.2)	1 (2.8)	11 (36.7)	0.001
Clinical outcomes				
Sepsis	44 (51.2)	17 (34.7)	27 (73)	0.001
Septic shock	25 (29.1)	5(10.2)	20 (54.1)	<0.001
Length of ICU stay, day (mean ± SD)	5.5 ± 7	3.9 ± 5.6	7.6 ± 8	0.021
Length of hospital stay, day (mean ± SD)	26.4 ± 31.4	18.2 ± 9.7	37.1 ± 44.7	0.016
Postoperative complications (C-D * ≥ 3)	24 (27.9)	5 (10.2)	19 (51.4)	<0.001
ICU mortality	9 (10.5)	2 (4.1)	7 (18.9)	0.035
7-day mortality	5 (5.8)	0	5 (13.5)	0.013
28-day mortality	8 (9.3)	1 (2)	7 (18.9)	0.019
In-hospital mortality	8 (9.3)	1 (2)	7 (18.9)	0.019
Overall mortality	9 (10.5)	2 (4.1)	7 (18.9)	0.035

EA, endotoxin activity. * Clavien–Dindo classification.

**Table 3:** Logistic regression analysis of risk factors for (A) postoperative complications and (B) 28-day mortality in patients after major abdominal surgery.

(A) Postoperative complications				
	Univariate analysis		Multivariate analysis	
	OR (95% CI)	*p*-value	OR (95% CI)	*p*-value
Age	1.021 (0.988–1.056)	0.219	-	-
EA level *	300.516 (16.149–5592.344)	<0.001	46.333 (1.001–847.027)	0.046
SOFA score *	1.522 (1.287–1.8)	<0.001	1.354 (1.065–1.72)	0.013
C-reactive protein *	1.258 (1.129–1.401)	<0.001	1.046 (0.946–1.157)	0.379
Lactate *	1.534 (1.234–1.908)	<0.001	1.006 (0.748–1.353)	0.968
(B) 28-day mortality				
	Univariate analysis		Multivariate analysis	
	OR (95% CI)	*p*-value	OR (95% CI)	*p*-value
Age	1.023 (0.97–1.079)	0.405	-	-
EA level *	30 (0.916–982.601)	0.05	1.673 (0.009–319.177)	0.848
SOFA score *	1.36 (1.135–1.63)	0.001	1.288 (1.028–1.613)	0.028
C-reactive protein *	1.066 (0.993–1.143)	0.078	-	-
Lactate *	1.367 (1.095–1.707)	0.006	1.103 (0.827–1.471)	0.503

EA, endotoxin activity; SOFA, sequential organ failure assessment. * Variables were measured at ICU admission after surgery.

**Table 4:** Comparison of clinical outcomes between two groups according to postoperative endotoxin activity (EA) levels in elevated total bilirubin (TB) group patients.

	Low EA (EA < 0.515)	High EA (EA ≥ 0.515)	*p*-Value
	n = 31 (66%)	n = 16 (34%)	
Clinical outcomes			
Sepsis	12 (38.7)	11 (68.8)	0.069
Septic shock	2 (6.5)	9 (56.3)	<0.001
Length of ICU stay, day (mean ± SD)	3.6 ± 6.1	9.8 ± 10.3	0.038
Length of hospital stay, day (mean ± SD)	18.6 ± 9.6	50 ± 61.6	0.06
Postoperative complications (C-D * ≥ 3)	2 (6.5)	10 (62.5)	<0.001
28-day mortality	1 (3.2)	4 (25)	0.04

EA, endotoxin activity. * Clavien–Dindo classification.

**Table 5:** Logistic regression analysis of risk factors for (A) postoperative complications and (B) 28-day mortality in patients after major abdominal surgery in elevated total bilirubin (TB) group patients.

(A) Postoperative complications				
	Univariate analysis		Multivariate analysis	
	OR (95% CI)	*p*-value	OR (95% CI)	*p*-value
Age	1.032 (0.986–1.08)	0.176	-	-
EA level *	10,286.214 (43.683–2,422,130.566)	0.001	613.85 (1.13–333,512.315)	0.046
SOFA score *	1.57 (1.239–1.99)	<0.001	1.328 (0.944–1.868)	0.013
C-reactive protein *	1.436 (1.156–1.783)	0.001	1.081 (0.895–1.307)	0.379
Lactate *	2.174 (1.372–3.445)	0.001	2.257 (0.842–6.051)	0.968
(B) 28-day mortality				
	Univariate analysis		Multivariate analysis	
	OR (95% CI)	*p*-value	OR (95% CI)	*p*-value
Age	1.023 (0.97–1.079)	0.405	-	-
EA level *	30 (0.916–982.601)	0.05	1.673 (0.009–319.177)	0.848
SOFA score *	1.36 (1.135–1.63)	0.001	1.288 (1.028–1.613)	0.028
C-reactive protein *	1.066 (0.993–1.143)	0.078	-	-
Lactate *	1.367 (1.095–1.707)	0.006	1.103 (0.827–1.471)	0.503

EA, endotoxin activity; SOFA, sequential organ failure assessment. * Variables were measured at ICU admission after surgery.

## Data Availability

All data analyzed and presented in this study are contained within this article. The raw data supporting the conclusions will be made available by the authors upon request.
